# Characterizing Sex Differences in Depressive-Like Behavior and Glial Brain Cell Changes Following Peripheral Nerve Injury in Mice

**DOI:** 10.3389/fnbeh.2021.758251

**Published:** 2021-10-29

**Authors:** Vassilia Michailidis, Navdeep K. Lidhar, Chulmin Cho, Loren J. Martin

**Affiliations:** ^1^Department of Cell and Systems Biology, University of Toronto, Toronto, ON, Canada; ^2^Department of Psychology, University of Toronto Mississauga, Mississauga, ON, Canada

**Keywords:** chronic pain, depressive-like, microglia, astrocytes, forced swim test and tail suspension test

## Abstract

Chronic pain and depression are intimately linked; the combination of the two leads to higher health care costs, lower quality of life, and worse treatment outcomes with both conditions exhibiting higher prevalence among women. In the current study, we examined the development of depressive-like behavior in male and female mice using the spared nerve injury (SNI) model of neuropathic pain. Males displayed increased immobility on the forced-swim test – a measure of depressive-like behavior – 2 weeks following injury, while females developed depressive-like behavior at 3-week. Since the pathogenesis of chronic pain and depression may involve overlapping mechanisms including the activation of microglial cells, we explored glial cell changes in brain regions associated with pain processing and affect. Immunohistochemical analyses revealed that microglial cells were more numerous in female SNI mice in the contralateral ventral anterior cingulate cortex (ACC), a brain region important for pain processing and affect behavior, 2-week following surgery. Microglial cell activation was not different between any of the groups for the dorsal ACC or nucleus accumbens. Analysis of astrocyte density did not reveal any significant changes in glial fibrillary acidic protein (GFAP) staining in the ACC or nucleus accumbens. Overall, the current study characterized peripheral nerve injury induced depression-like behavior in male and female mice, which may be associated with different patterns of glial cell activation in regions important for pain processing and affect.

## Introduction

Chronic pain is one of the most prevalent and debilitating conditions affecting as many as 20% of the population worldwide (Goldberg and McGee, 2011). It is the foremost cause of long-term sick leave, and disability imposing a profound health care burden that not only affects the individual but also permeates throughout all avenues of social, work, and family life (Lynch, 2011; Gaskin and Richard, 2012). However, despite the prevalence of chronic pain, it remains challenging to treat, in part because it is associated with a high incidence of comorbid conditions, including anxiety and depression (Nicholson and Verma, 2004). Approximately 50% of patients diagnosed with major depressive disorder reportedly suffer from chronic pain, and the risk of developing comorbid depression in chronic pain patients is greater among women (Radat et al., 2013). Further, patients suffering from chronic pain and depression have a poorer prognosis than patients suffering from each illness alone (Arnow et al., 2006; Munce and Stewart, 2007). Clinically, it is well known that chronic pain and depression are related; however, the neurobiological changes responsible for the co-occurrence of these conditions are not well-understood, and the sex-specific mechanisms that might help explain the high prevalence of these conditions in women remain unknown.

Microglia, a principal immune cell, comprise approximately 5–12% of the glial cell population and emerge from either erythromyeloid precursors of the embryonic yolk sac or invade the central nervous system (CNS) via myeloid progenitors and proliferate during embryonic and postnatal development (Hristovska and Pascual, 2015). Microglia interact with and prune synapses during healthy brain development to modify their structure and function (Chen et al., 2018b). As such, in a healthy resting state, microglia actively sense the environment to maintain normal physiological conditions; however, following tissue damage or inflammation, microglia transition from an inactive ramified state to an active amoeboid state – characterized by enlarged cell bodies and shortened processes and increased expression of microglial markers such as ionized calcium-binding adapter molecule 1 (IBA-1) and CD11b (Ohsawa et al., 2004; Ji et al., 2013; Chen et al., 2018b). A few studies have demonstrated that chronic pain hypersensitivity is dependent on microglia in male but not female rodents (Sorge et al., 2015; Chen et al., 2018b; Mapplebeck et al., 2018). Further, spinal microglial activation promotes BDNF release and pain hypersensitivity in male but not female rats – an effect that is conserved in humans (Dedek et al., 2021).

An abundance of research has focused on spinal mechanisms; however, there is evidence to suggest that brain microglia may contribute to the development and maintenance of chronic pain and its associated comorbidities. A study by Miyamoto et al. (2017) showed that intracerebroventricular injections of minocycline – a broad-spectrum antibiotic known to inhibit glial cell activity – reduced microglial activation in the anterior cingulate cortex (ACC) and reversed tactile allodynia. In a rat model of diabetic neuropathy, ammoxetine, a novel, potent serotonin, and norepinephrine reuptake inhibitor, reversed mechanical allodynia and depressive-like behavior that coincided with decreased spinal microglial activation (Zhang et al., 2018). Furthermore, a recent study using a rodent model of neuropathic pain, called chronic constriction injury (CCI), revealed that male mice developed affective disorders, albeit at a delayed time point, which was associated with activation of microglia characterized by increased numbers and cell body size in the prefrontal cortex, amygdala, and hippocampus (Barcelon et al., 2019).

There is also evidence to suggest that brain astrocytes may be involved in regulating pain sensitivity and affect following injury (Chen et al., 2018b). Several studies have demonstrated that activated astrocytes in the ACC and hippocampus are involved in the maintenance of chronic pain and the onset of depression (see Li et al., 2019 for a review). Narita et al. (2006) demonstrated that peripheral nerve injury in mice increased glial fibrillary acidic protein (GFAP) expression in the ACC, which was associated with increased affective behaviors. Similarly, Ikeda et al. (2013) used a mouse model of inflammatory pain and showed increased astrocytic expression in the ACC resulted in increased affective behavior that was attenuated with the administration of L-α-aminoadipate (L-AA), an astroglia toxin. Further, the administration of antidepressant drugs inhibits hippocampal astrocyte activation and reduces nerve injury-induced pain hypersensitivity (Zhu et al., 2009). However, there seems to be a paucity of research on astrocytes in chronic pain and depression, necessitating further investigation.

The primary goal of the present study was to identify whether sex influenced the development of depressive-like behavior in mice following spared nerve injury (SNI). A secondary goal was to characterize whether brain glial activation was increased in a time-, sex-, and-, brain region-dependent manner following injury. Based off clinical studies showing a greater degree of comorbid depression in female chronic pain patients (Munce and Stewart, 2007), we hypothesized that female mice would display greater and more robust signs of depressive-like behavior, which would be associated with increased glial activity. We used a battery of behavioral tests and examined two early time points (2 and 3 weeks) following nerve injury. Microglia and astroglia in the ACC (ventral and dorsal compartments) and the nucleus accumbens were quantified given data supporting a role for these regions in clinical anhedonia and response to treatment (Pizzagalli et al., 2001; Wacker et al., 2009; Carl et al., 2016).

## Materials and Methods

### Mice

Outbred CD-1 male and female mice (6–8 weeks old) obtained from Charles River were used and maintained in the University of Toronto Mississauga’s Animal Care Facility. All mice were housed in non-ventilated cages in groups of 2–4, maintained in a temperature-controlled (20 ± 1°C) environment with 12:12 h light: dark cycle (lights on at 7 am and off at 7 pm) with access to food (Harlan Teklad 8604) and water *ad libitum*. Experiments were conducted only during the light period. All procedures were performed in accordance with the guidelines of the Canadian Council on Animal Care and approved by the University of Toronto Animal Care Committee.

### Behavioral Measures

The automated von Frey, forced swim test (FST), tail suspension test (TST), open field, and sucrose preference assays were used on three independent cohorts of mice. In all experiments, mice were habituated to the testing environment for at least 1 h before the experiment. The first cohort was used to assess mechanical sensory thresholds and forced swim behavior at the indicated time points. Following baseline von Frey measurement, mice were randomly assigned to either the SNI or sham group and the 2-week or 3-week post-surgery timepoint groups. FST was only tested once for each animal on its respective timepoint (i.e., at 2-week post-surgery or 3-week post-surgery). If a mouse was assigned to the 3-week time point, its mechanical threshold was measured at 2-week post-surgery and 3-week post-surgery, while FST was only measured at 3-week post-surgery. Following the FST, mice were sacrificed, and brain tissue was collected for immunohistochemical analysis. The second cohort was tested for tail suspension and open-field behavior at the indicated time points, while a third cohort was tested for sucrose preference weekly.

#### Automated von Frey

An automated von Frey test (Ugo Basile Dynamic Plantar Aesthesiometer) was used to assess mechanical nociceptive thresholds in all mice. Mice were placed in custom-constructed Plexiglas cubicles (6.3 cm × 5.5 cm × 10 cm) on a perforated metal floor and allowed to habituate for 1 h before testing. A blunt probe was raised toward the plantar surface of the hind paw, upon which pressure was gradually increased until the mouse withdrew its hind paw; the maximal pressure displayed at that point was then recorded. Five consecutive measures were taken on both hind paws. In all experiments, von Frey measurements were taken in both ipsilateral and contralateral hind paws before surgery (baseline), 2-week, and 3-week post-surgery. However, only data from the hind paw ipsilateral to the injury are presented as no significant differences on the contralateral paws were observed.

#### Spared Nerve Injury

The spared nerve injury (SNI) model, an experimental nerve injury, was used to produce neuropathic pain in mice (Decosterd and Woolf, 2000). Mice were randomly assigned to one of two surgery groups, sham or SNI. Briefly, mice were anesthetized with isoflurane (4% for induction; 2.5% for maintenance), and the biceps femoris muscle was bluntly dissected to expose the sciatic nerve. The common peroneal and tibial branches of the sciatic nerve were ligated with silk sutures (7.0 silk, Ethicon), and a 1 mm portion of the nerve was removed below the suture, leaving the sural nerve intact. The muscle and skin were then stitched with sutures (6.0 coated vicryl, Ethicon). The sham surgery consisted of a similar blunt dissection of the biceps femoris muscle without dissection of the nerve. Mice were allowed to recover in their home cages for 2 weeks following surgery.

#### Forced Swim Test

The forced swim test was used as a measure of behavioral despair. All mice were placed in glass cylinders (25 cm × 14.6 cm) filled with water (24 ± 1°C) 15 centimeters deep for 6 min and recorded using standard Sony video recording devices. Solid brown dividers were placed between each glass container to prevent mice from observing each other. Videos were uploaded to a tracking software (EthoVision, Noldus) which automatically calculated the time spent immobile in seconds.

#### Tail Suspension Test

In a subset of mice, the tail suspension test was used as a measure of learned helplessness. This test monitors the amount of time spent immobile when suspended by the tail over 6 min. The mouse was securely fastened by the end of the tail to a flat surface that was suspended in a Plexiglas box (40 cm × 40 cm × 40 cm). Behavior was recorded using a video camera and analyzed for immobility using Noldus EthoVision.

#### Open Field Test

Mice were placed in a Plexiglas box (40 cm × 40 cm × 40 cm) and videotaped for offline analysis. The open field was divided into a 4 by 4 grid. The four center squares in the grid were considered the “open area,” while the 12 perimeter boxes were analyzed as “wall areas.” All videos were recorded and analyzed using Noldus EthoVision.

#### Sucrose Preference Test

Mice were habituated to a 2% sucrose solution in their home cage (150 ml) for 2 days. Two bottles were then attached to the home cages of individually housed mice to ensure sucrose preference before starting the experiment. Mice were then randomly divided into the sham or SNI condition. Mice were then given free access to two bottles, one containing a 2% sucrose solution (150 ml) and the other water (150 ml) for 24 h. Following the 24 h interval, the amount of liquid consumed was then measured, and sucrose preference was calculated as the average of the daily amount of sucrose solution consumed divided by the total liquid consumed from both bottles. Food and water were restricted for 17 h (4:00 pm – 9:00 am), preceding a two-bottle choice as previously done (Chu et al., 2016). During the 2-bottle choice, mice had free access to food.

### Immunohistochemistry

Following behavioral testing, mice were deeply anesthetized with pentobarbital and transcardially perfused with cold phosphate buffered saline (PBS) and 4% paraformaldehyde (PFA). Immediately following transcardial perfusions, whole brains were isolated and post-fixed in 4% PFA for 4 h at 4°C and then cryoprotected in 30% PBS-sucrose until sectioning. Brains were hemisected along the sagittal axis and then sectioned using a cryostat (−13°C to −20°C) into 40 μm coronal sections and stored in tissue storage buffer until needed for staining. Sections were washed with PBS for 5 min and then washed in 0.1% PBS-T three times for 5 min. Slices were blocked with goat serum for 2 h and washed three times for 5 min in 0.1% PBS-T. Finally, slices were incubated using two primary antibodies [Anti-Iba1, Red Fluorochrome (635)-conjugated, Wako Chemical; and Anti-GFAP, Cy3 Conjugate, Millipore Sigma] for 48 h at 4°C. Following a post-incubation period of 48 h, slices were washed with PBS for 5 min and then mounted onto Superfrost slides and imaged using the Cytation 5 Cell Imaging Multimode Reader (BioTek, Winooski, VT, United States) at 20x objective. Image acquisition settings were identical for all slices across all time points. Negative controls omitting the primary antibody resulted in a complete absence of positive staining. Immunohistochemistry was run in batches of slices with sections from each group/condition included in each run.

### Glial Cell Quantification

Microglia and astrocytes were measured in the contralateral and ipsilateral dACC (*n* = 5–6/sex/timepoint), vACC (*n* = 5–6), and Nac (*n* = 5–6). Briefly, microglia numbers were imaged and counted across three brain slices per region of interest (ROI) for each hemisphere. Images were taken using the Cytation 5 Imaging Multi-Mode Reader (BioTek, Winooski, VT, United States) at 4X for regional identification and 20X for cell counting. For each slice, a z-stack projection of 40 μm was generated, and each region of interest was outlined and measured using Gen5 software (BioTek, Winooski, VT, United States). Cells expressing fluorescence were counted manually by an experimenter who was blind to experimental conditions using the same area settings for each brain region. Cells were considered microglia based on colocalization between Iba-1 and DAPI. Astrocytes were quantified by measuring GFAP fluorescence signal in the ROI using the GEN5 software. GFAP intensity was calculated to be the GFAP-integrated intensity minus the background-integrated intensity for each ROI. All GFAP images were acquired using the same exposure settings. In all instances, the individual performing this analysis was blind to the condition.

### Statistical Analyses

SPSS Statistics 24 (IBM, Chicago, IL, United States) software was used to perform all analyses using three- or four-way ANOVAs as appropriate. Unless otherwise stated, a *p*-value of less than 0.05 was considered statistically significant. *Post hoc* testing was performed using paired or independent *t*-tests between sham and SNI mice within sex and time point.

## Results

### Depressive- and Anxiety-Like Behavior Is Expressed Differently in Male and Female Mice Following Spared Nerve Injury

**FIGURE 1:**
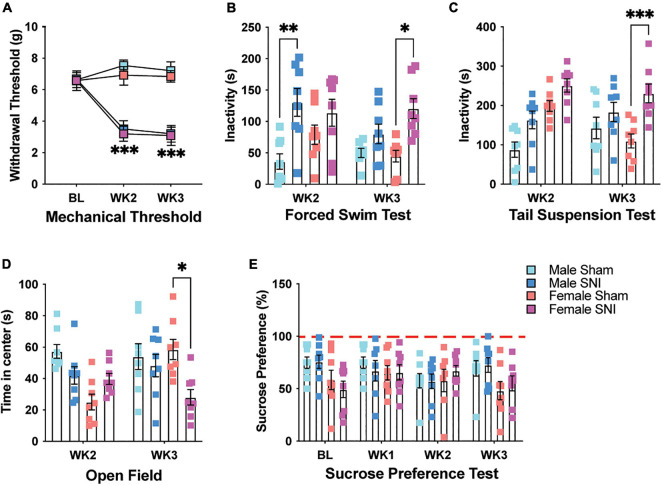
SNI induces mechanical sensitivity and depressive-like behavior. **(A)** Mechanical allodynia is evident in male and female mice following SNI, but not sham surgery, 2- and 3-week post-injury (*n* = 16 for BL and WK2; *n* = 8 for WK3/sex/condition). **(B)** SNI increases immobility on the forced swim test in male mice 2-week post-surgery, while immobility increases in female SNI mice 3-week post-surgery (*n* = 8/sex/condition). **(C)** SNI increases immobility on the tail suspension test in female mice 3-week post-surgery (*n* = 8/sex/condition). **(D)** Female SNI mice spend less time in the center area of the open field 3-week post-surgery (*n* = 8/sex/condition). **(E)** SNI did not alter sucrose preference in male and female mice at any of the tested timepoints (*n* = 7–8/sex/condition). *p < 0.05; **p < 0.001; ***p < 0.0001.

To determine the effect of chronic pain on depressive-like behavior in mice, we used the SNI model of neuropathic pain (Decosterd and Woolf, 2000). Following surgery, SNI mice demonstrated a significant increase in hind paw mechanical hypersensitivity compared with sham controls when tested 2- and 3-week post-surgery [three-way repeated measures (RM) ANOVA, main effect of surgery: *F*_1_,_28_ = 262.45, *p* < 0.001; main effect of sex: *F*_1,28_ = 3.47, *p* = 0.08; main effect of time (RM): *F*_2,27_ = 30.79, *p* < 0.001; surgery × time interaction: *F*_2,27_ = 75.28, *p* < 0.001, Figure 1A]. There was no difference in hypersensitivity between male and female SNI mice (surgery × sex interaction: *F*_1,28_ = 0.007, *p* > 0.05). To determine whether neuropathic pain altered behavioral despair, mice were tested for immobility on the forced swim test (FST). Male and female mice with SNI displayed overall greater immobility on the FST. However, immobility was greater for male SNI mice 2-week following surgery, while female SNI mice showed more immobility 3-week following surgery when compared with sham controls (three-way ANOVA, main effect of surgery: *F*_1,55_ = 27.29, *p* < 0.001; main effect of sex: *F*_1,55_ = 1.868, *p* = 0.17; main effect of time: *F*_1,55_ = 2.01, *p* = 0.16; surgery × sex × time interaction: *F*_1,55_ = 5.42, *p* = 0.02; Figure 1B). Using a separate cohort of mice, we used the tail suspension test (TST) to determine whether the effects of SNI on male and female mice extrapolated to another measure of behavioral despair. In line with the results of our FST, SNI mice displayed greater immobility on the TST with female mice showing greater signs of behavioral despair 3-week following surgery (three-way ANOVA, main effect of surgery: *F*_1,56_ = 23.52, *p* < 0.001; main effect of sex: *F*_1,56_ = 12.72, *p* < 0.001; main effect of time: *F*_1,56_ = 0.57, *p* = 0.32; sex × time interaction: *F*_1,56_ = 9.56, *p* = 0.003; Figure 1C). This same cohort of mice was also tested for time spent in the center of the open field as a general measure of anxiety. Female SNI mice showed reduced time spent in the center area of the open field 3-week following surgery, indicating an anxiety-like phenotype at this time point (three-way ANOVA, main effect of surgery: *F*_1,56_ = 5.92, *p* = 0.018; main effect of sex: *F*_1,56_ = 6.95, *p* = 0.011; main effect of time: *F*_1,56_ = 1.33, *p* = 0.25; surgery × sex × time interaction: *F*_1,56_ = 8.44, *p* < 0.01; Figure 1D). Walking distance in the open field was not significantly different between the groups indicating that differences on these tests were not caused by mobility issues (three-way ANOVA, All *F*_1,56_ values < 1.88, and all *p* values > 0.17; *data not shown*). Finally, we tested a third cohort of mice on the sucrose preference assay to measure whether SNI induced a state of anhedonia in male and female mice. SNI did not alter sucrose preference in male or female mice following surgery. There was only a slight sex difference with male mice displaying a greater sucrose preference (three-way ANOVA, main effect of surgery: *F*_1_,_26_ = 0.003, *p* = 0.95; main effect of sex: *F*_1,2__6_ = 4.24, *p* = 0.05; main effect of time: *F*_3_,_78_ = 1.187, *p* = 0.32; Figure 1E).

### Microglial Expression in the Ventral Anterior Cingulate Cortex Is Different Between the Sexes Following Spared Nerve Injury

**FIGURE 2:**
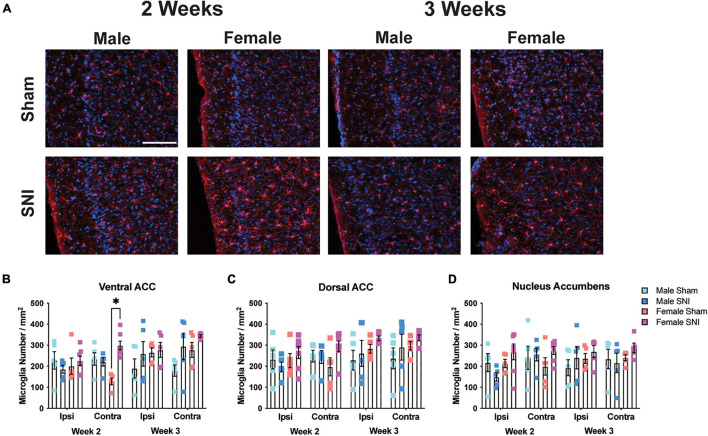
Microglia changes in selected brain regions following SNI. **(A)** Representative fluorescent micrographs taken from the contralateral ventral ACC for male and female mice across surgery condition and timepoints. Scale bar = 100 μM. **(B)** SNI increases microglial number in the contralateral ventral ACC 2-week post-surgery. Microglia remained elevated in female mice 3-week post-surgery but was no longer significant from the sham condition. SNI did not alter microglia numbers in the **(C)** dorsal ACC, or **(D)** nucleus accumbens (*n* = 5–6/sex/condition, three tissue samples per mouse). *p < 0.05.

Next, to understand whether the expression of microglia in brain regions associated with depressive-like behavior may be associated with the sex-specific effects on the FST and TST, we examined microglial cell numbers in the dorsal and ventral ACC, and nucleus accumbens. To analyze microglia cell number in each brain structure, four-way mixed ANOVAs [hemisphere (RM) × surgery × sex × timepoint] were conducted on each region. For the ventral ACC, microglial cells were more numerous in the contralateral (i.e., right) hemisphere in female SNI mice compared with sham female mice at the 2-week timepoint [main effect of hemisphere (RM): *F*_1,34_ = 1.26, *p* = 0.26; main effect of surgery: *F*_1,34_ = 7.28, *p* = 0.011; main effect of time: *F*_1,34_ = 6.06, *p* = 0.02; main effect of sex: *F*_1,34_ = 2.71, *p* = 0.11; hemisphere (RM) × condition × sex × time interaction: *F*_1,34_ = 4.43, *p* = 0.043; Figures 2A,B]. However, analysis of the dorsal ACC did not reveal any significant effects [main effect of hemisphere (RM): *F*_1,34_ = 3.52, *p* = 0.07; main effect of surgery: *F*_1,34_ = 1.86, *p* = 0.18; main effect of sex: *F*_1,34_ = 2.17, *p* = 0.14; main effect of time: *F*_1,34_ = 3.35, *p* = 0.08; Figure 2C]. Finally, analysis of the nucleus accumbens revealed an effect of hemisphere [main effect of hemisphere (RM): *F*_1,34_ = 4.38, *p* = 0.044; main effect of surgery: *F*_1,34_ = 1.001, *p* = 0.32; main effect of sex: *F*_1,34_ = 1.71, *p* = 0.2; main effect of time: *F*_1,34_ = 0.29, *p* = 0.59; hemisphere × surgery × sex × time interaction: *F*_1,34_ = 3.38, *p* = 0.074; Figure 2D].

### Astrocyte Density in Male and Female Mice Following Spared Nerve Injury

**FIGURE 3:**
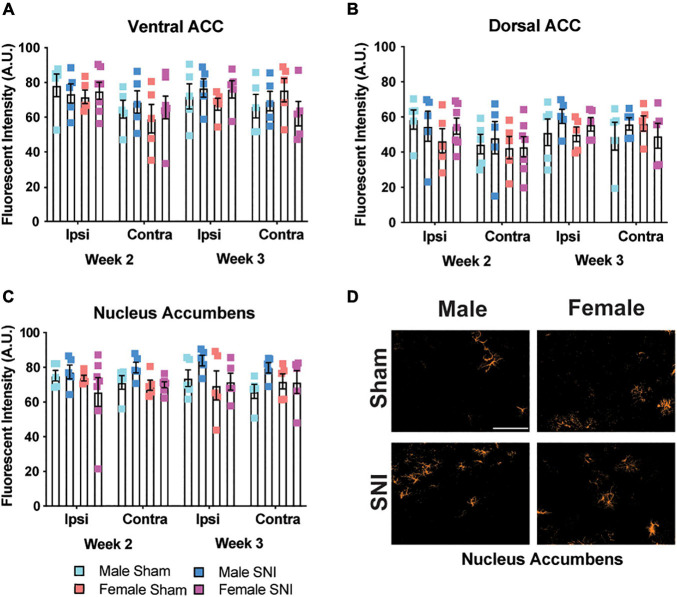
Astrocyte changes in selected brain regions following SNI. Astrocyte expression is not changed following SNI in the **(A)** ventral ACC, **(B)** dorsal ACC, or **(C)** nucleus accumbens in a time and sex-dependent manner (*n* = 5–6/sex/condition, three tissue samples per mouse). **(D)** Representative images taken from the contralateral nucleus accumbens show astrocyte expression for male and female mice at the 2-week time point. Fluorescence is presented as arbitrary units.

Since astrocytes have been shown to contribute to depressive-like behavior (Zhang et al., 2020) and are increased in the brain following nerve injury (Zhu et al., 2009), we measured the fluorescence intensity of GFAP^+^ staining in the same brain regions as microglial analysis. Fluorescence intensity was used for astrocyte quantification due to their larger size and lack of clear definition, which made it difficult to determine whether branches were from one astrocyte or several. There was a significant hemispheric effect for the ventral ACC with greater intensity of GFAP+ staining in the ipsilateral hemisphere that was independent of surgery condition (four-way ANOVA, main effect of hemisphere: *F*_1,34_ = 8.11, *p* < 0.01; main effect of surgery, *F*_1,34_ = 0.2, *p* = 0.66; main effect of sex: *F*_1,34_ = 0.038, *p* = 0.54; main effect of time: *F*_1,34_ = 0.09, *p* = 0.76, Figure 3A). We did not observe any significant effects for GFAP staining in the dorsal ACC (four-way ANOVA, all *F*’s < 1.499; all *p*’s < 0.23, Figure 3B). In the nucleus accumbens, there was a slight main effect for sex with males exhibiting overall higher GFAP staining and an interaction between surgery condition and sex that was due to higher GFAP staining in male SNI mice (four-way ANOVA, main effect of hemisphere: *F*_1__,34_ = 0.36, *p* = 0.55; main effect of surgery: *F*_1,34_ = 1.83, *p* = 0.18; main effect of sex: *F*_1,34_ = 4.23, *p* = 0.04; main effect of time: *F*_1,34_ = 0.06, *p* = 0.8; surgery × sex interaction: *F*_1,34_ = 4.379, *p* = 0.044; Figures 3C,D).

## Discussion

The current study used a mouse model of chronic neuropathic pain and investigated the development of depressive-like behavior in male and female mice. We also characterized changes in microglia number and astrocyte density as a measure of activation following injury. Overall, there were sex differences in the onset of depressive-like behaviors in male and female mice such that males displayed immobility on the forced swim test at an earlier time point than females, and only female mice developed immobility on the tail suspension task following nerve injury. In the contralateral vACC, microglia cell number was significantly greater for female SNI mice than sham. No other changes in glial cells were apparent.

The onset of the depressive-like phenotype was different in male and female mice, which mirrors the human literature on the prevalence of depression and shows different onset rates for each sex (Romans et al., 2007). In our study, males developed the depressive phenotype 2-week following injury, while females developed depressive- and anxiety-like behavior at 3-week, suggesting that initially, females may be more resilient than males, or changes in affect may take longer to manifest in females. However, depressive-like behaviors have been shown to manifest in male mice at later time points (i.e., 8-week following injury) when the chronic constriction injury model of neuropathic pain was used (Barcelon et al., 2019). We do not believe that the effects of nerve injury on FST immobility are related to movement because nerve injury did not affect walking or general movement in the OFT. However, the possibility exists that depending on sex, behavioral tests such as the FST and TST might be more sensitive for the detection of subtle behavioral differences. As another behavioral measure, we assessed anhedonia using the sucrose preference test; however, nerve injury did not alter sucrose intake. We do not believe that the lack of effect for the sucrose preference test was related to our testing procedures because during pilot studies sucrose intake was decreased when we used a positive control (i.e., restraint stress, *data not shown*). Anhedonia is challenging to induce in mice, and various pain models fail to cause a robust change in sucrose preference following injury (Schwartz et al., 2014). The sucrose preference test did reveal lower baseline preference for female than male mice. This is interesting because there have been reported sex differences in sucrose preference, but they typically are in the opposite direction with female rodents showing a greater preference (McCall et al., 2013). However, a few studies have shown that behavioral taste responses to dilute sucrose solutions are decreased by estrogen, whereas ovariectomy abolishes this effect (Curtis et al., 2004). This suggests that higher levels of estrogen in female mice may elevate the threshold for gustatory detection of sweet taste, a possibility that has been previously suggested (Curtis et al., 2005). Thus, sex differences in this assay may depend on estrogen-modulation of taste responses to specific concentrations of sucrose in these testing procedures.

Human studies are far more complicated than the current mouse experiments and the prevalence of comorbid depression with pain may depend on how and where the patients were assessed and the criteria for depression used, such as severity, assessment method and sample selection (Okifuji and Turk, 2016). While many studies have examined the prevalence of comorbid pain and depression (Tunks et al., 2008; Rayner et al., 2016), few studies fail to specifically examine coping strategies when patients suffer from both conditions as well as recovery time for pain and/or depression. A human study by Rovner et al. (2017) demonstrated that although the severity of chronic pain was the same for females and males, females were more accepting of the pain, remained more active, and reported fewer mood disturbances than males, supporting the idea that initially, females are better able to cope with chronic pain. In humans, females are more likely to endorse rumination (Meints et al., 2017) and rely on social support (Rovner et al., 2017) than males. These coping strategies may be the result of female patients reporting greater levels of pain dismissal (Igler et al., 2017), which may contribute to delayed-onset depression. Unfortunately, no study using a human population has examined whether there is a sex-difference in the relationship between chronic pain duration and the onset of depression. Thus, it is possible that female patients are likely to cope with pain better than males, but when coping strategies fail, such as lack of social support the consequence may be increased prevalence of depression.

In addition, pain severity, pain duration and number of pain locations are associated with the recurrence of depressive and anxiety disorders (Gerrits et al., 2014; Sharpe et al., 2017). To our knowledge, no single study has examined whether male patients recover from comorbid depression quicker than females; however, inflammatory markers are predictive of depression in men, but not in women suggesting that the etiology of depressive disorders within the patient population are different between genders (Ernst et al., 2021). In our study, increased inflammation may have contributed to the early behavioral signs of depression in male mice, however, we did not measure general inflammation at the 2-week time point and cannot be certain that this was the precise mechanism. Further, male mice recovered from the depressive phenotype at 3-week, suggesting that males may adapt to chronic pain and eventually recover from affect-related disorders. These results are in line with studies in mice (Sellmeijer et al., 2018) and may explain why there is a higher incidence of depression in women with chronic pain than men (Munce and Stewart, 2007).

While several research groups have used microglial cell body size to quantify microglia activation (Taylor et al., 2017; Guneykaya et al., 2018), we used microglial cell number as a less subjective metric (Barcelon et al., 2019). In the contralateral vACC female SNI mice displayed significantly more microglia cell bodies than sham controls whereas SNI mice did not display overall changes in astrocyte intensity. Previous research has indicated that sex differences exist for microglia in the brain, but following nerve injury, the degree of microglia and astrocyte activation is similar between the sexes (Chen et al., 2018a). A possible explanation for this is that following nerve injury, cortical microglia may not be activated in the same way that spinal microglia have been characterized (Coull et al., 2005). Recent evidence suggests that there is an interaction between the spinal dorsal horn and the ACC in pain modulation, but mechanisms in the spinal cord do not necessarily transfer to cortical mechanisms (Tsuda et al., 2017). Previous reports characterizing brain microglia have mainly used CCI-induced nerve injury in male mice, suggesting that injury type may impact microglial or astrocyte activation in the brain differently between the sexes (Taylor et al., 2017).

We used Iba1 and GFAP as molecular markers for activated microglia and astrocytes, respectively, because of their roles in demonstrating morphological features at different microscopic levels (Shapiro et al., 2008; Sofroniew and Vinters, 2010). However, limitations do exist with these two markers and should be noted. Iba1 is not only a marker for activated microglia but also other macrophages that are recruited during nerve injury as well as other subpopulations of microglia (i.e., ramified microglia) (Ohsawa et al., 2004; Shapiro et al., 2009). So, Iba1 immunohistochemical expression may not be limited to just activated microglia and thus may affect analysis and interpretation of results. It is challenging to acquire quantitative data with the IHC techniques used in the current paper. While some, but not all papers complement their IHC results with qPCR for verification, we did not do this and is a limitation of the present study. In addition, GFAP does not always label healthy CNS astrocytes (Sofroniew and Vinters, 2010), which could alter the difference observed in astrocyte density between nerve-injured and control mice. Further, we considered using Sholl analysis to capture astrocyte complexity; however, in our staining the larger size of the astrocytes and their lack of clear definition, made it difficult to determine whether branches were from one astrocyte or many. The center point of many astrocytes was also not always evident, making other quantification methods difficult. Thus, it would be important to consider the use of complementary techniques or other activated microglia and astrocyte markers, such as TMEM-119 (a transmembrane protein found only on microglia) (Bennett et al., 2016) and SOX9 (expressed exclusively by astrocytes) (Sun et al., 2017), respectively. These markers may be better suited for detecting active glia states and understanding reactive changes in glial cells.

Since, spinal nociceptive afferents are expected to innervate the contralateral side of the brain, our analysis considered whether lateralization occurred in any of the brain regions following injury as previously shown (Taylor et al., 2017). Our results stand in contrast to Taylor et al. (2017), where robust lateralization and regional differences were uncovered; however, we did find a lateralization effect for the number of microglia in the vACC of female SNI mice. Here, microglia cell number per square millimeter was significantly greater in SNI versus sham mice. Notably, there were minimal changes in microglia, and astrocytes even though several other studies have shown microglia changes in the thalamus, amygdala, ventral tegmental area (VTA), nucleus accumbens, bed nucleus of the stria terminalis, and periaqueductal gray following peripheral nerve injury (Taylor et al., 2015; Ni et al., 2016; Liu et al., 2017). Given that we found minimal differences, we did not pursue a mechanistic line of inquiry and we do not know whether glial changes in the ventral ACC are related to the depressive-like behavior in female SNI mice. As with some of our behavioral results, comparing between the 2- and 3-week time points is difficult because there may be inherent baseline expression differences between the mice. There may also be inherent sex differences in microglial expression as previously shown with male microglia being more numerous in the cortex, hippocampus, and amygdala (Guneykaya et al., 2018).

Further investigations may want to explore whether microinjections of glial inhibitors into either the ipsilateral or contralateral hemisphere reverse the pain or depressive phenotype. In line with this, a previous study showed that microglia and astrocytes were increased in the ACC of nerve injured mice and microinjections of minocycline into the contralateral ACC partially reversed mechanical allodynia (Cooper et al., 2018). However, the side of nerve injury (i.e., left vs. right) plays a big role in whether functional pain responses are altered by brain region and hemispheric manipulations. For instance, inactivation of the right or bilateral central amygdala (CeA) attenuates mechanical allodynia and hyperalgesia when SNI is performed on the left side of the body, while inactivation of the left CeA has no effect. The same paper also showed that following right-sided SNI, mechanical allodynia was attenuated only by inactivation of the left CeA, while mechanical hyperalgesia was reduced by left, right and bilateral inactivation of the CeA (Cooper et al., 2018). There is also evidence showing that overproduction of interleukin-1β, a cytokine that activates microglia is a common mechanism underlying the generation of neuropathic pain, memory deficits, and depressive-like behavior in mice (Gui et al., 2016). Thus, a potential strategy may be to target upstream activators of microglia, rather than focus on direct glial inhibition.

Overall, the most important aspect of the current study was the demonstration that SNI induced depressive- and anxiety-like behavior differently in male and female mice. However, this study encourages further research on comorbid pain and depression using both sexes as there are clear behavioral sex differences and understanding the sex-specific mechanisms should be further explored.

## Data Availability Statement

The raw data supporting the conclusions of this article will be made available by the authors, without undue reservation.

## Ethics Statement

All procedures were performed in accordance with the guidelines of the Canadian Council on Animal Care and approved by the University of Toronto Animal Care Committee.

## Author Contributions

LM and VM conceived and designed the experiments and wrote the article. VM and CC performed the experiments. LM supervised the acquisition of results. VM, NL, and LM analyzed the data. All authors edited and commented on the final version of the manuscript.

## Conflict of Interest

The authors declare that the research was conducted in the absence of any commercial or financial relationships that could be construed as a potential conflict of interest.

## Publisher’s Note

All claims expressed in this article are solely those of the authors and do not necessarily represent those of their affiliated organizations, or those of the publisher, the editors and the reviewers. Any product that may be evaluated in this article, or claim that may be made by its manufacturer, is not guaranteed or endorsed by the publisher.
